# Genomic evidence of genetic diversity and functional evolution in *Flavobacterium columnare*

**DOI:** 10.3389/fmicb.2023.1240471

**Published:** 2023-09-28

**Authors:** Rui Han, Yuhao Hong, Ruilong Xu, Wenjie Guo, Mingshu Zhang, Zijun Lu, Qing Han, Zequan Mo, Xueming Dan, Yanwei Li

**Affiliations:** ^1^University Joint Laboratory of Guangdong Province, Hong Kong and Macao Region on Marine Bioresource Conservation and Exploitation, College of Marine Sciences, South China Agricultural University, Guangzhou, China; ^2^Nansha-South China Agricultural University Fishery Research Institute, Guangzhou, China; ^3^School of Bioscience and Bioengineering, South China University of Technology, Guangzhou, China; ^4^Guangdong Key Laboratory of Animal Conservation and Resource Utilization, Guangdong Public Laboratory of Wild Animal Conservation and Utilization, Institute of Zoology, Guangdong Academy of Sciences, Guangzhou, China

**Keywords:** *Flavobacterium columnare*, single-gene phylogeny, comparative genomics, cgMLST, genome-wide analysis (GWA)

## Abstract

*Flavobacterium columnare* is the causative agent of columnaris disease in freshwater fish. Columnaris disease can cause heavy economic losses in aquaculture. In this study, whole-genome sequencing was used to characterize this pathogen. *F. columnare* isolate AH-01 had a circular chromosome and plasmid that encoded a total of 3,022 genes. Isolate GX-01 only had a circular chromosome and encoded 2,965 genes. Genomic islands, prophage regions, and CRISPR/Cas systems were identified in both genomes. Both genomes presented evidence of gene variation and horizontal transfer, both of which are the essential components of genetic diversity, genome plasticity, and functional evolution. Single-gene phylogeny and comparative genome analyses were performed to investigate the variation and evolution of this pathogen. Genetic analysis of 16S rRNA and housekeeping gene sequences significantly clustered 55 *F. columnare* isolates into four clades. The intragroup identity of the 16S rRNA gene exceeded 99%, while the intergroup identity was below the species delineation threshold. We discovered significant translocation, inversion, and rearrangement events that influenced local synteny within each group. Notably, the observed alignments varied considerably among all the studied groups. The core genomes of all strains with available sequences comprised 747 genes, corresponding to approximately 25% of the genome. Core genome multilocus sequence typing, genome-wide orthology and phylogenetic analyses, and average nucleotide identity suggested that the currently existing *F. columnare* was an assemblage of several distinct species, with levels of divergence at least equivalent to those between recognized bacterial species. The present investigation provided genomic evidence of gene variation and horizontal transfer, which were the basis of genetic diversity, genome plasticity, and functional evolution. The findings supported a proposed new taxonomic perspective on *F. columnare*.

## 1. Introduction

Columnaris disease is a serious diseas affecting a variety of freshwater fish, including carp, grass carp, channel catfish, salmonids, black mollies, eels, goldfish, perch, tilapia, and others (Decostere et al., 1998, 1999, 2002; Suomalainen et al., 2006; Soto et al., 2008; Declercq et al., 2013). Columnaris disease may develop acutely in young fish and induce acute, subacute, or chronic infection in adults (Pacha and Ordal, 1967). This disease can cause massive skin lesions and gill or fin necrosis (Morrison et al., 1981; Decostere, 2002), which extends from the base of the dorsal fin and wraps around it, forming a shape resembling the back of a horse saddle (Pacha and Ordal, 1967; Morrison et al., 1981). Hence, the disease is also known as “saddle-back disease”. Columnaris disease results in high mortality rates in fish, which produce huge economic losses in the aquaculture industry worldwide (Bullock et al., 1986).

*Flavobacterium columnare* is the causative agent of columnaris disease. It was first isolated in 1922 and was initially named *Bacillus columnaris*. It was subsequently classified into the family *Flavobacteriaceae* (Davis, 1922; Bernardet et al., 1996). *F. columnare* is a long Gram-negative rod with gliding motility that forms yellow-pigmented and smooth or rhizoid colonies (Bernardet et al., 1996; Kunttu et al., 2009; Declercq et al., 2013). Given the severity and rapid spread of columnaris disease, *F. columnare* has been studied for many years, especially its genetic diversity. Many molecular genotyping approaches can be used to determine genetic diversity, such as random amplified polymorphic DNA (RAPD), pulsed-field gel electrophoresis (PFGE), 16-23S intergenic spacer region (ISR) sequencing, amplified fragment length polymorphism (AFLP), single-strand conformation polymorphism (SSCP), and others. Restriction fragment length polymorphism of the 16S rRNA gene (16S-RFLP) is a method that classifies *F. columnare* isolates into distinct “genomovars”. 16S-RFLP was once considered to be standard for typing *F. columnare* (Song et al., 1988; Triyanto and Wakabayashi, 1999; Arias et al., 2004; Darwish and Ismaiel, 2005; Olivares-Fuster et al., 2007; LaFrentz et al., 2014; Garcia et al., 2018). More recently, *F. columnare* isolates were reclassified into four genetic groups by using multilocus phylogenetic analysis (MLPA), which may be more scientifically rigorous (LaFrentz et al., 2018). The simple relationship between these two classification methods is as follows: genetic group 1 corresponds to genomovar I, genetic groups 2 and 4 correspond to genomovar II, and genetic group 3 corresponds to genomovar III. More recently, a polyphasic approach was proposed to confirm the phylogenetic relationships of *F. columnare*; the previous genetic groups 2, 3, and 4 were divided into a new species in the genus *Flavobacterium* (LaFrentz et al., 2022).

The complete genome sequence of an organism can be considered the ultimate genetic map. Comparative genomics can identify unique genes of different species, and reveal differences in nucleotide composition, collinearity, pathogenicity, and host tropism of different pathogens. Although there are 33 complete *F. columnare* genomes in the GenBank database, the available data are limited because some of the genomes are phage-infected variants of FCO-F2 and FCO-F9, while others are incompletely assembled. To date, there are only a few analyses of the genomes or comparative genomes of flavobacterial pathogens and even fewer of *F. columnare* (Kayansamruaj et al., 2017; Kumru et al., 2017, 2020; Tekedar et al., 2017; Zhang et al., 2017).

In 2018 and 2019, we obtained 45 *F. columnare* isolates from diseased grass carp (*Ctenopharyngodon idella*) in 11 provinces of China (Lu et al., 2021). In 2020, we isolated an additional 10 strains at the South China Agricultural University breeding base (Table 1). This study involved a genetic analysis of a total of 55 preserved *F. columnare* strains. From these strains, we selected two (AH-01 and GX-01) for whole-genome sequencing. Additionally, we performed a comparative genome analysis on 16 *F. columnare* strains to obtain comprehensive insights into their pathogenic mechanisms, phylogenetic relationships, and taxonomic status. Finally, we explored the internal connections and differences among *F. columnare* strains and propose novel perspectives concerning *F. columnare* evolution and classification.

**Table 1:** Description of the 10 isolates obtained from South China Agricultural University.

**Strain**	**Tissue source**	**Isolate number**
Gi-01, Gi-02, Gi-03, Gi-04	Gill	4
Mu-03, Mu-04	Muscle	2
L-05, L-06	Liver	2
SP-05	Spleen	1
K-06	Kidney	1

## 2. Materials and methods

### 2.1. Bacterial strains

Fifty-five *F. columnare* isolates (Table 1) (Lu et al., 2021) for DNA extraction and genome sequencing were cultured on modified Shieh agar plates and in Shieh broth with shaking at 28°C at 200 rpm (Decostere et al., 1997).

### 2.2. DNA extraction, PCR amplification, and phylogenetic analyses

Bacterial genomic DNA (gDNA) of the 55 *F. columnare* isolates was extracted using an E.Z.N.A.^®^ Bacterial DNA Kit (catalog no. D3350; Omega Bio-tek, Norcross, GA, USA). The housekeeping genes *gyrB, tuf*, and *dnaK* were amplified by PCR using PrimeSTAR^®^ MaxDNA polymerase (TaKaRa Bio, Shiga, Japan). A PCR mix (25 μL) containing 1 μL of gDNA template, 12.5 μL of high-fidelity polymerase, 1 μL of forward primer, 1 μL of reverse primer, and 9.5 μL of distilled water was used for sequencing, using previously described primers and cycling protocols (LaFrentz et al., 2018).

Sequence alignment was performed with the BLAST website (https://blast.ncbi.nlm.nih.gov/Blast.cgi) and the ClustalW2 program (http://www.ebi.ac.uk/Tools/clusta-lw2/index.html), and a phylogenetic tree was constructed using Molecular Evolutionary Genetics Analysis (MEGA) software (ver. 7.0) with the neighbor-joining method and 1,000 bootstrap replications.

### 2.3. Genome sequencing and assembly

Genomic DNA of *F. columnare* strains AH-01 and GX-01 isolated from Anhui Province and Guangxi Province in China, respectively, was extracted with the sodium dodecyl sulfate method (Lim et al., 2016), detected using agarose gel electrophoresis, and quantified using a Qubit^®^ 2.0 Fluorometer (Thermo Fisher Scientific, Waltham, MA, USA). Whole genomes of *F. columnare* AH-01 and GX-01 were sequenced using the PacBio Sequel platform and Illumina NovaSeq PE150 at the Beijing Novogene Bioinformatics Technology Co., Ltd. To ensure the accuracy of the subsequent analysis, low-quality reads (<500 bp) were filtered out to obtain clean data. Long reads (>6,000 bp) were selected as seed sequences using the automatic error correction function of the Single-molecule real-time (SMRT) portal. Shorter reads were aligned to the seed sequences using blasr for preliminary assembly. The arrow algorithm was then used to correct and count variant sites in the preliminary assembly results using the variant calling module of SMRT Link software (https://www.pacb.com/support/software-downloads/). The corrected assembly used as the reference sequence was subjected to a BLAST search against Illumina data. Whether or not the chromosomal sequence formed a circle was determined, and the initial site was corrected by a BLAST search against the DNAa database based on the overlap between the head and tail. Subsequently, chromosome and plasmid sequences were screened by BLAST against a plasmi database (http://plasmidb.sourceforge.net/). Circos software was used to display the genome according to the assembled genome sequence combined with the prediction of coding genes (Krzywinski et al., 2009).

### 2.4. Genome feature analysis and component prediction

A whole-genome BLAST search using Diamond (E-value <1e-5) was performed against the Gene Ontology (GO) (Ashburner et al., 2000), Kyoto Encyclopedia of Genes and Genomes (Kanehisa et al., 2004, 2006; KEGG), Clusters of Orthologous Groups (COG; Galperin et al., 2015), Non-Redundant Protein (NR; Li et al., 2002), and Swiss-Prot (Bairoch and Apweiler, 2000) databases to predict gene functions. Meanwhile, The IslandViewer4 program (Bertelli et al., 2017), which integrates three different identification approaches, was used to retrieve genomic islands (GIs). To predict prophages, the sequence was examined using the default settings of the PHASTER web server (Arndt et al., 2016). CRISPRminer2 [including three methods: CRISPRCasFinder, CRISPR Recognition Tool (CRT), and PILER-CR] was used for the identification of the CRISPR system (Zhang et al., 2018).

### 2.5. Comparative genome analysis

Fourteen publicly available genomes in NCBI were downloaded for genome comparisons with AH-01 [CP097867-CP097868] and GX-01 [CP097869]: ATCC 49512 [NCBI accession no. GCA_000240075.2], Pf1 [GCA_001677395.1], TC 1691 [GCA_001936395.1], FCO-F2 [GCA_014844255.1], FCO-F9 [GCA_014844775.1], ATCC 23463 [GCA_002530675.1], AL-02-36 [GCA_019565575.1], C#2 [GCA_001641185.1], 94-081 [GCA_001534645.1], ARS1 [GCA_004010215.1], 1215 [GCA_002204815.1], 90-106 [GCA_019565505.1], Costa Rica 04-02-TN [GCA_019565455.1], and NK01 [GCA_002204895.1]. To reconstruct a phylogenetic tree, *Flavobacterium johnsoniae* UW101 [GCA_000016645.1] and *F. psychrophilum* JIP02/86 [GCA_000064305.2] were also used as outgroup clades.

The “Bacterial Pan Genome Analysis” (BPGA; Chaudhari et al., 2016) pipeline is software for calculating the pan-genome and core genome, which are calculated iteratively based on exponential growth and decay models for every sequential addition of the genome of a new strain. We used this model to predict the core pan-genome of *F. columnare* with a default setting. The protein homologs were clustered by USEARCH with an identity cutoff value of 50%. Core genome multilocus sequence typing (cgMLST) was performed based on the core genome sequences, and MEGA software (ver. 7.0) was used to construct a phylogenetic tree with the neighbor-joining method and 1,000 bootstrap replications.

MAUVE genome alignment software (Darling et al., 2004) was used with default settings for genome collinearity analysis. Orthology analysis was performed using OrthoVenn2 (Xu et al., 2019) and OrthoFinder (Emms and Kelly, 2015). The Type (Strain) Genome Server (TYGS) pipeline was used to reconstruct a phylogenetic neighbor-joining tree, which was inferred using FastME 2.1.4 from genome BLAST distance phylogeny (GBDP) distances calculated based on genome sequences (Lefort et al., 2015; Meier-Kolthoff and Goker, 2019). To determine general genetic similarity, a pairwise comparison of average nucleotide identity (ANI) was performed using the recommended default settings of the JSpeciesWS Online Service (Richter et al., 2016) and TBtools software for image processing (Chen et al., 2018); Digital DNA–DNA hybridization (dDDH) analysis was performed using the Genome-to-Genome Distance Calculator (GGDC) 3.0 (Meier-Kolthoff et al., 2022).

## 3. Results

### 3.1. Genetic analysis of 55 *F. columnare* isolates

We first analyzed the 16S rRNA and housekeeping gene sequences of the 55 F. columnare isolates stored in our lab and constructed a phylogenetic tree using MEGA software.

#### 3.1.1. 16s rRNA gene sequence similarity and single-gene phylogeny

**Figure 1:**
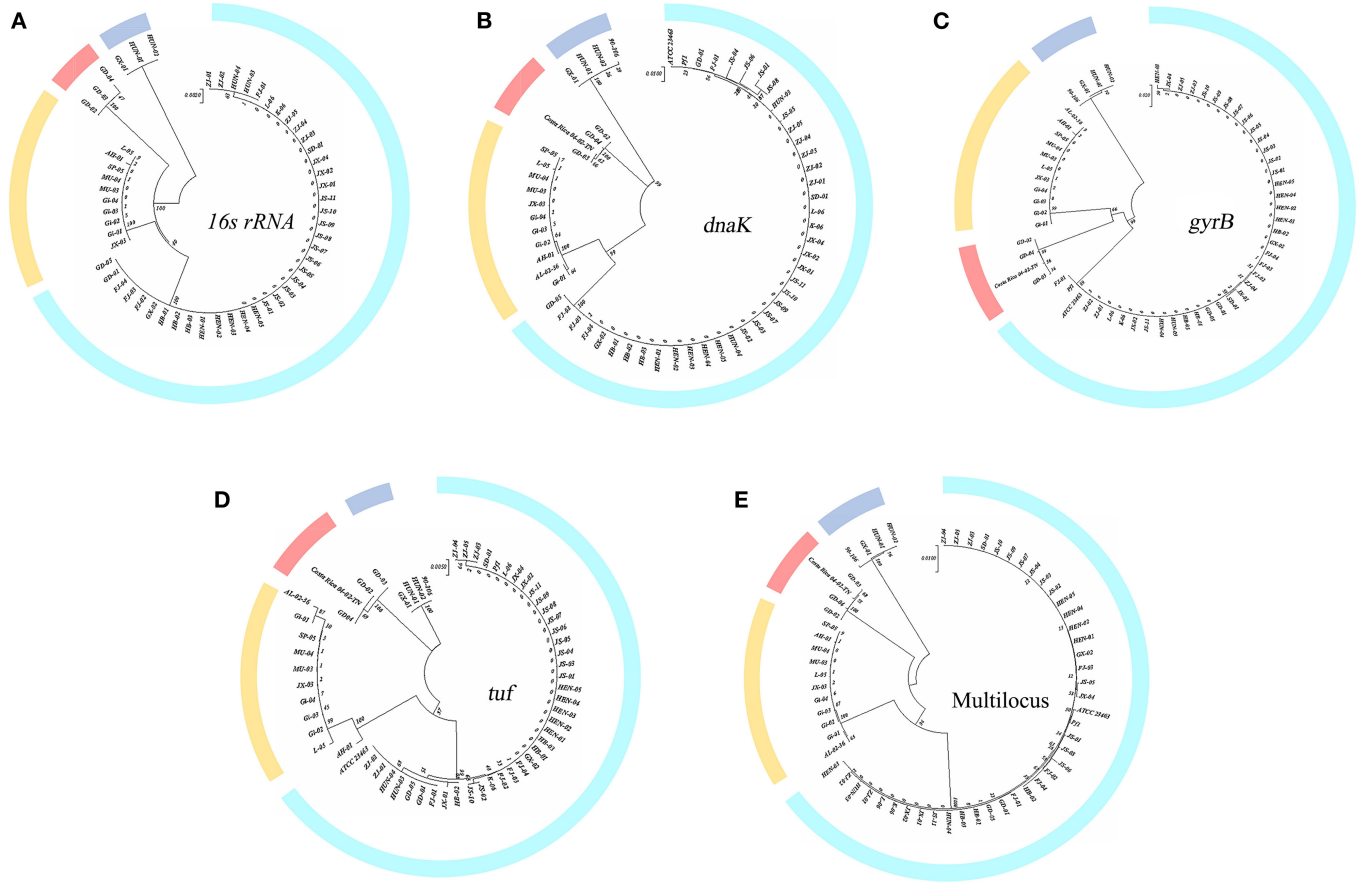
Single-gene phylogenetic tree construction. **(A)** 16S rRNA gene; **(B–D)** *dnaK, gyrB*, and *tuf* gene. **(E)** Multilocus tree of concatenation of single housekeeping gene.

Figure 1A shows the phylogenetic trees constructed based on 16S rRNA gene sequences, which divided these strains into four clusters. The 16S rDNA sequences used in this article have been uploaded to GenBank (accession numbers: MW548534-MW548578 and OR064175-OR064184).

The percent identity of the 16S rRNA gene among these isolates was roughly divided into four groups, which also correspond exactly to the groups of previous studies (LaFrentz et al., 2018). The percent identity of the 16S rRNA gene within the group exceeded 99%, while the values among groups did not. The 16S rRNA gene similarity between genetic groups 1 and 2 reached >98%, between genetic groups 1 and 4 was >97.8%, and between genetic groups 1 and 3 was 97% (genetic groups 2 and 4 both belong to genomovar II).

#### 3.1.2. Housekeeping gene sequence similarity and phylogenetic analysis

Figures 1B, D were constructed based on the housekeeping genes *dnaK* and *tuf* . These two figures showed that the genetic group 1 and 2 strains clustered into one branch, and each was a subclade. Genetic group 3 and 4 strains clustered into one branch; the phylogenetic tree of *gyrB* in Figure 1C reveals a slight difference. Strains of genetic groups 1, 2, and 4 were first divided into a large branch and distinguished from genetic group 3. Then, genetic groups 2 and 4 were separated from genetic group 1, with genetic groups 2 and 4 forming a group of their own. Figure 1E was obtained by the concatenation of single genes. Similar to Figure 1A, the strains of genetic group 3 formed a branch, while genetic groups 1 and 2 were clustered into a subclade and separated from genetic group 4 (genetic groups 2 and 4 both belong to the previously defined genomovar II). The sequence identities of three housekeeping genes (*gyrB, tuf*, and *dnaK*) are listed separately in Supplementary Table 1. Consistent with the results of the 16S rRNA gene, four groups were formed.

### 3.2. Genomic characterization of AH-01 and GX-01

To expand the genome data of *F. columnare* isolated from China for subsequent comparative analysis, we selected AH-01 and GX-01 for genome sequencing. They belonged to genetic groups 2 and 3, respectively, and were highly virulent.

#### 3.2.1. Annotation of predicted genes

The complete genome of *F. columnare* strain AH-01 consisted of a circular chromosome and a circular plasmid with an average G+C content of 31.3%. The genome size of AH-01 is 3,407,076 bp (including a 704.31 Kb plasmid), which encodes a total of 3,022 genes with 31 rRNA, 87 tRNA, and 3 sRNA genes. Strain GX-01 had a single 3,438,997-bp circular chromosome with an average G+C content of 31.05%. This encoded 2,965 genes, with 36, 93, and 3 rRNA, tRNA, and sRNA genes, respectively. Geographical origin, tissue source, genome size, contig number, G+C content, and other features of AH-01 and GX-01 are listed in Table 2.

**Table 2:** Genome features of *F. columnare* strains AH-01 and GX-01.

	**AH-01**	**GX-01**
Geographical origin	Anhui (CHN)	Guangxi (CHN)
Tissue source	Gill	Spleen
Size (bp)	3,407,076	3,438,997
Contig number	2	1
Plasmid	1	0
%GC	30.86/31.67	31.05
Total genes	3,022	2,965
Average gene size (bp)	970	988
%Coding region	86.01	85.18
rRNA	31	36
tRNA	87	93
sRNA	3	3

**Figure 2:**
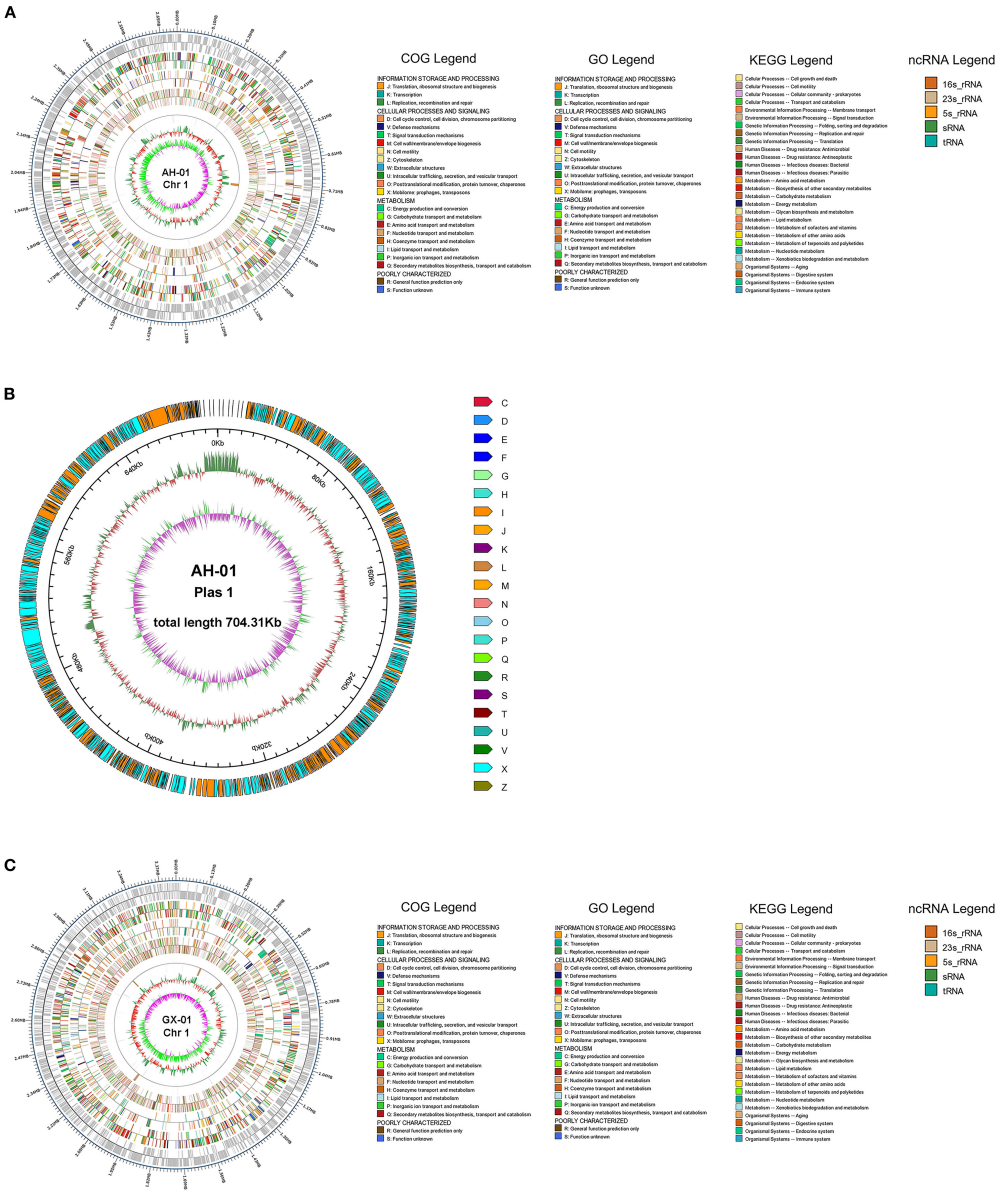
Visualizing genomic Circos map of *F. columnare* strains AH-01 and GX-01. From the outside to the inside circle, there are genome position coordinates, coding genes, gene function annotation results (including COG, GO, and KEGG database annotation results), ncRNA, genome GC content (The inward red part indicates that the GC content of the region is lower than the average GC content of the whole genome, while the outward green part is opposite of the red part, and the higher value means the greater difference from the average GC content), and genome GC skew value (The specific algorithm is G-C/G+C). The inward pink part indicates that the G content in this area is lower than the C content, and the outward light green part has the opposite meaning). **(A)** A Circos map of AH-01 chromosome 1. **(B)** AH-01 plasmid 1. **(C)** GX-01 chromosome 1.

We used several databases to annotate predicted genes. The protein sequences of these genes were compared against each functional database using Diamond (E-value ≤ 1e-5). AH-01 had 1,806 genes assigned to COG, 1,745 to GO, 2,681 associated with KEGG, and 2,554 matched in NR-*Flavobacterium columnare*. GX-01 had 1,820 genes assigned to COG, 1,769 to GO, 2,594 to KEGG, and 2,388 matched in NR (Supplementary Figures 1, 2). Most AH-01 and GX-01 genes were classified in the same sets of GO, KEGG, and COG. One gene from AH-01 was uniquely classified in cell killing, and one gene from GX-01 was classified in rhythmic processes in GO. Furthermore, Circos software (Krzywinski et al., 2009) was used to display genome maps of *F. columnare* AH-01 and GX-01 strains based on the assembled genome sequences combined with the predicted coding genes (Figure 2).

#### 3.2.2. Genome component prediction and function analysis

**Figure 3:**
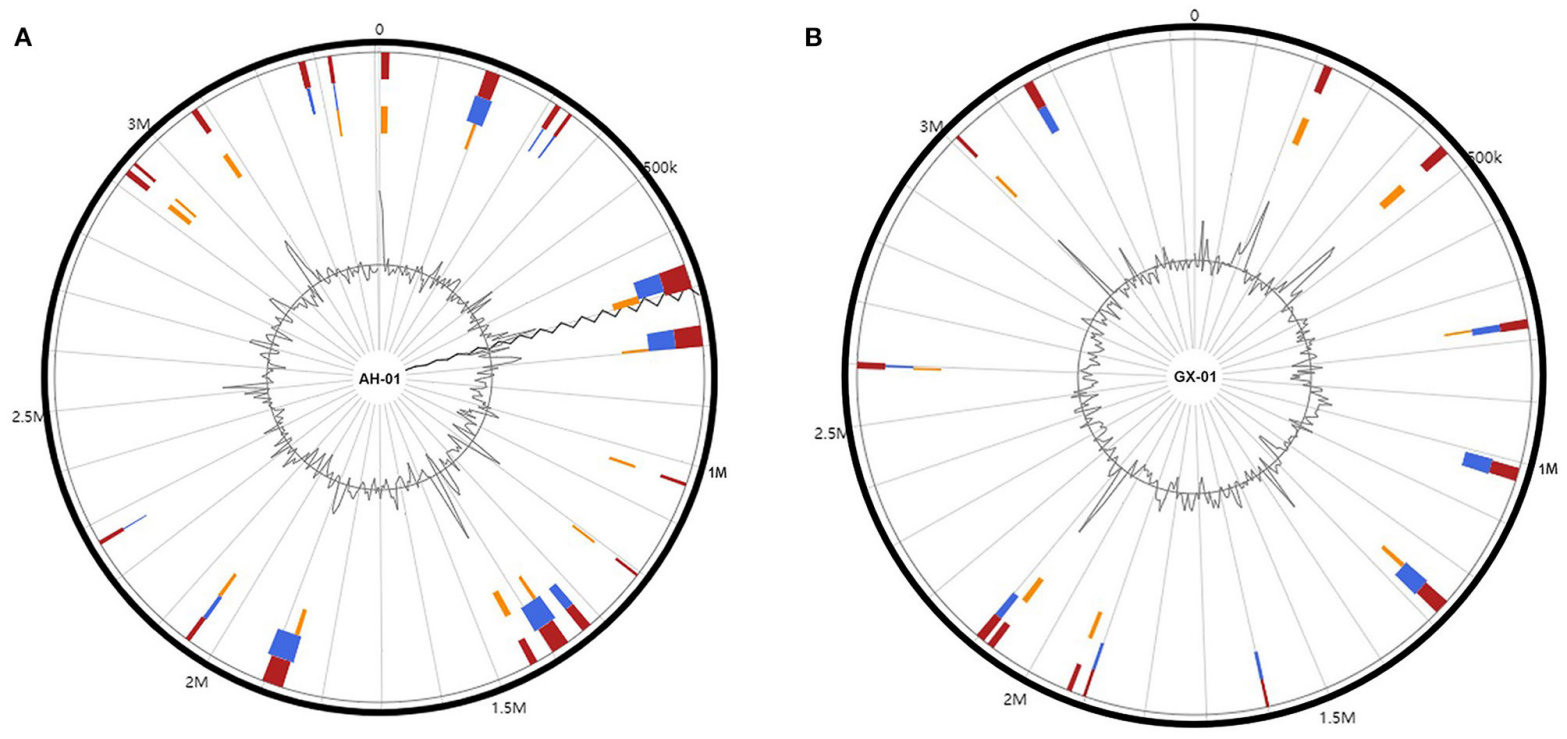
Genomic islands in the genomes of **(A)** AH-01 and **(B)** GX-01. Red: integrated prediction methods; orange: SIGI-HMM prediction methods; blue: IslandPath-DIMOB prediction method.

To further investigate the genetic diversity of these strains, we used the IslandViewer4 pipeline to identify GIs using more than one prediction method (Figure 3). Nineteen and thirteen integrated GIs were identified in AH-01 and GX-01, respectively, by more than one prediction method (Figure 3). These predicted GIs comprised mostly hypothetical proteins, transposases, integrases, and transcriptional regulators. The adenosine triphosphases associated with diverse cellular activities (AAA) family ATPases were found in both strains. Furthermore, strain AH-01 had some unique proteins in its GIs, such as glycosyl hydrolase, serine hydrolase, serine/threonine protein kinase, and a transporter protein, while strain GX-01 had some unique proteins, such as metallo-beta-lactamase fold metallohydrolase, chloramphenicol acetyltransferase, GTP-binding protein, and other proteins. Additional GI information is provided in Supplementary Table 3.

**Figure 4:**
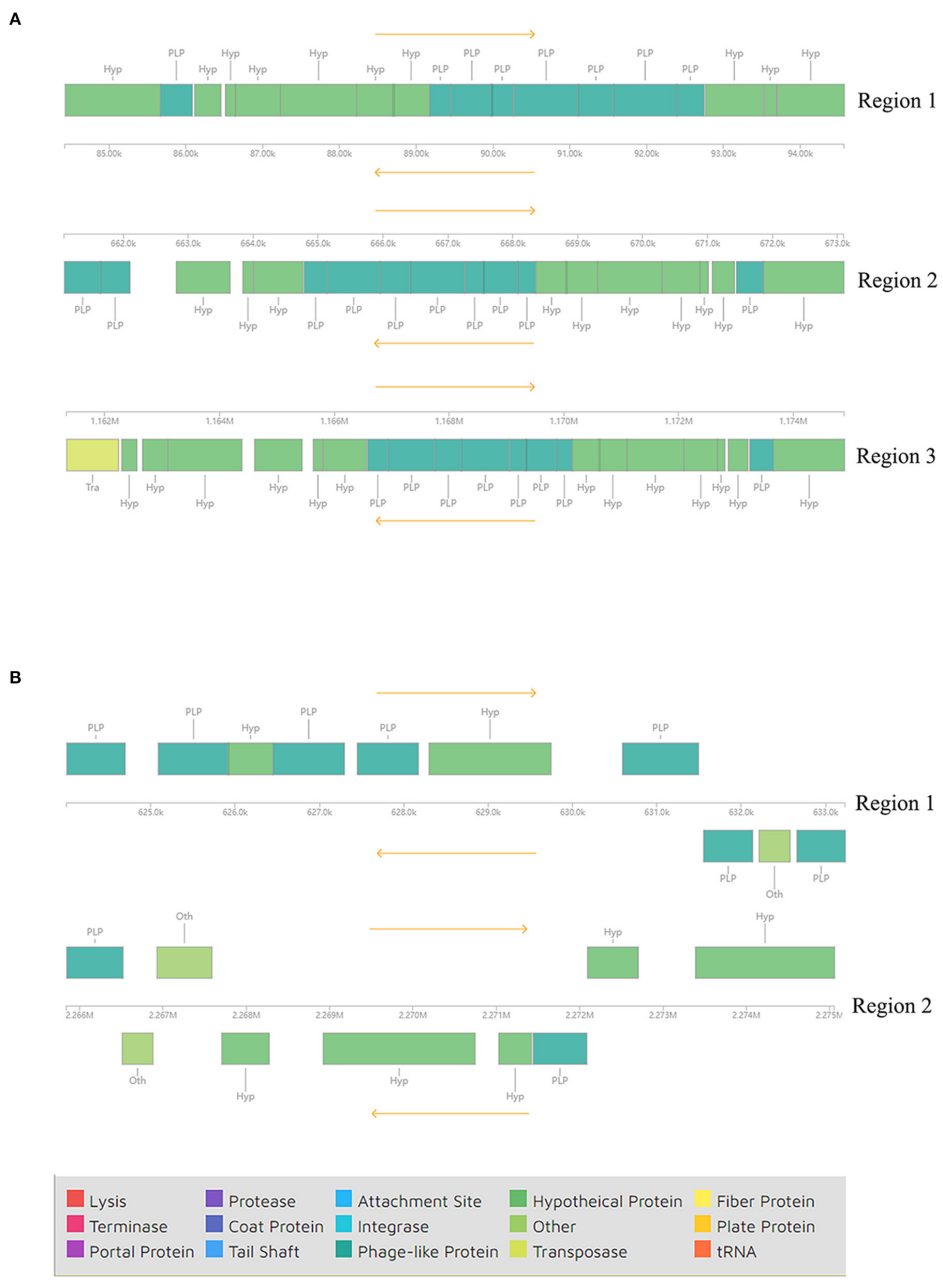
Components of incomplete phages in **(A)** AH-01 and **(B)** GX-01 were identified by the PHASTER tool.

In addition to GIs, we also identified prophage regions in the genomes of these strains using PHASTER. Three incomplete prophage regions were identified in different locations of strain AH-01 with a length of 10.1–13.5 kb. Proteins in regions 1 and 2 were hypothetical proteins and phage-like proteins, while a transposase protein was also present in region 3 (Figure 4A). However, strain GX-01 carried two incomplete prophage regions, both 9.2 kb. Hypothetical proteins, phage-like proteins, and other proteins existed in all regions (Figure 4B). Supplementary Table 4 provides more details.

**Figure 5:**
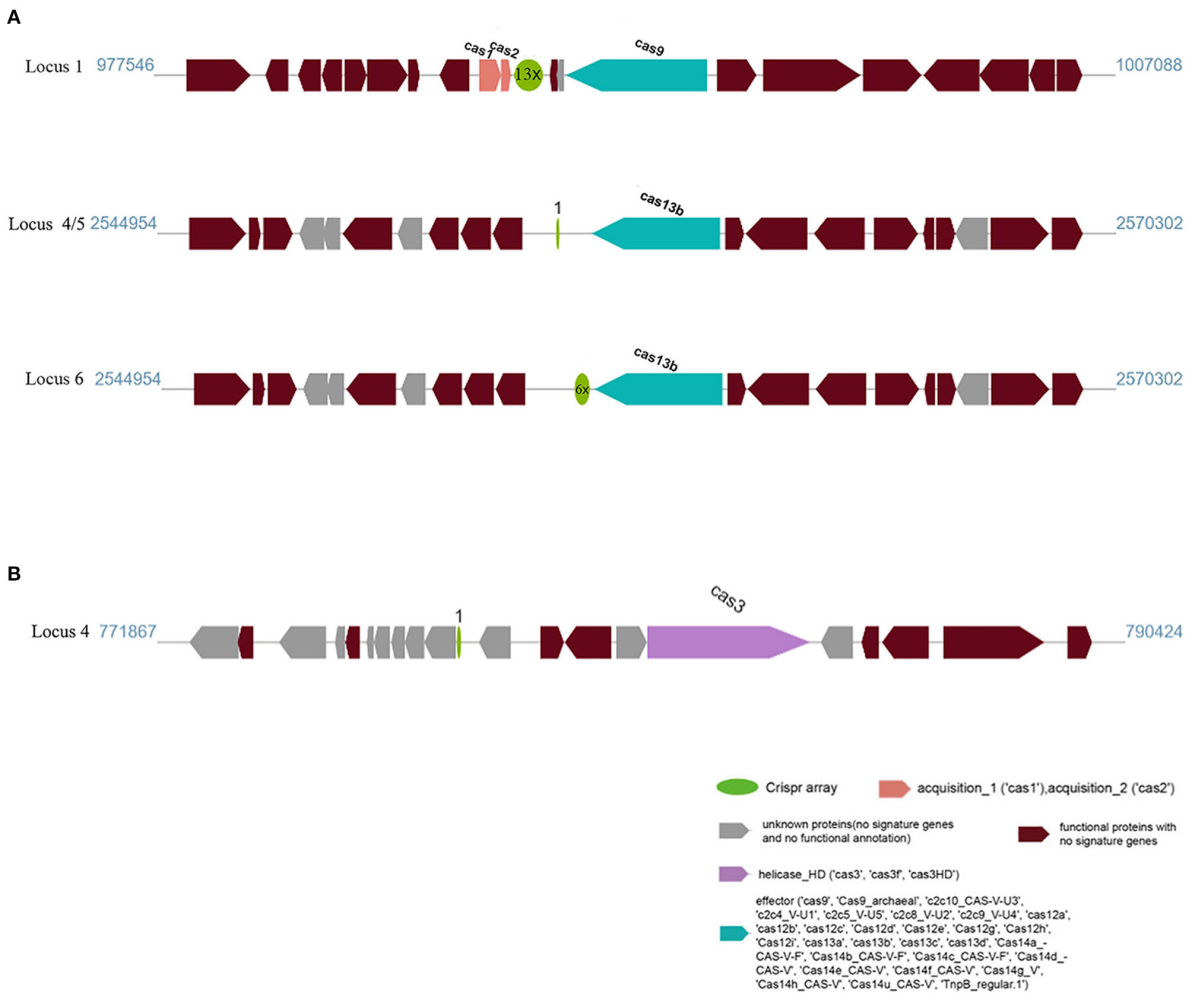
CRISPR locus present in **(A)** AH-01 and **(B)** GX-01. The dark red blocks represent functional proteins, and the green circles mean CRISPR arrays containing direct repeat sequences with repeat times; the spacer was not shown in the figure. The adjacent Cas genes were also marked in blue, pink, and purple, respectively.

Another feature of the genomes of these strains was the presence of a CRISPR/Cas system. We used the CRISPRminer2 web tool to predict the CRISPR arrays and *cas* genes. Six CRISPR arrays were identified on the AH-01 chromosome. Only loci 1, 4, 5, and 6 were linked to Cas protein-associated genes (*cas1, cas2*, and *cas13b*). The other loci contained only a CRISPR array without *cas* genes nearby. The CRISPR types of loci 1, 4, 5, and 6 were Type II, VI-B1, and VI-B2. By contrast, strain GX-01 had only one locus with *cas3* nearby, and the other five arrays were orphan CRISPRs lacking *cas* genes. A schematic diagram of CRISPR loci is shown in Figure 5. CRISPR locations, consensus repeats, and other information are provided in Supplementary Table 5.

### 3.3. Comparative genomic analysis of 16 *F. columnare* strains

To perform comparative genomic analysis, we included 14 well-assembled strains from GenBank, in addition to AH-01 and GX-01. These 16 strains belong to different genetic groups.

#### 3.3.1. Core pan-genome analysis

**Figure 6:**
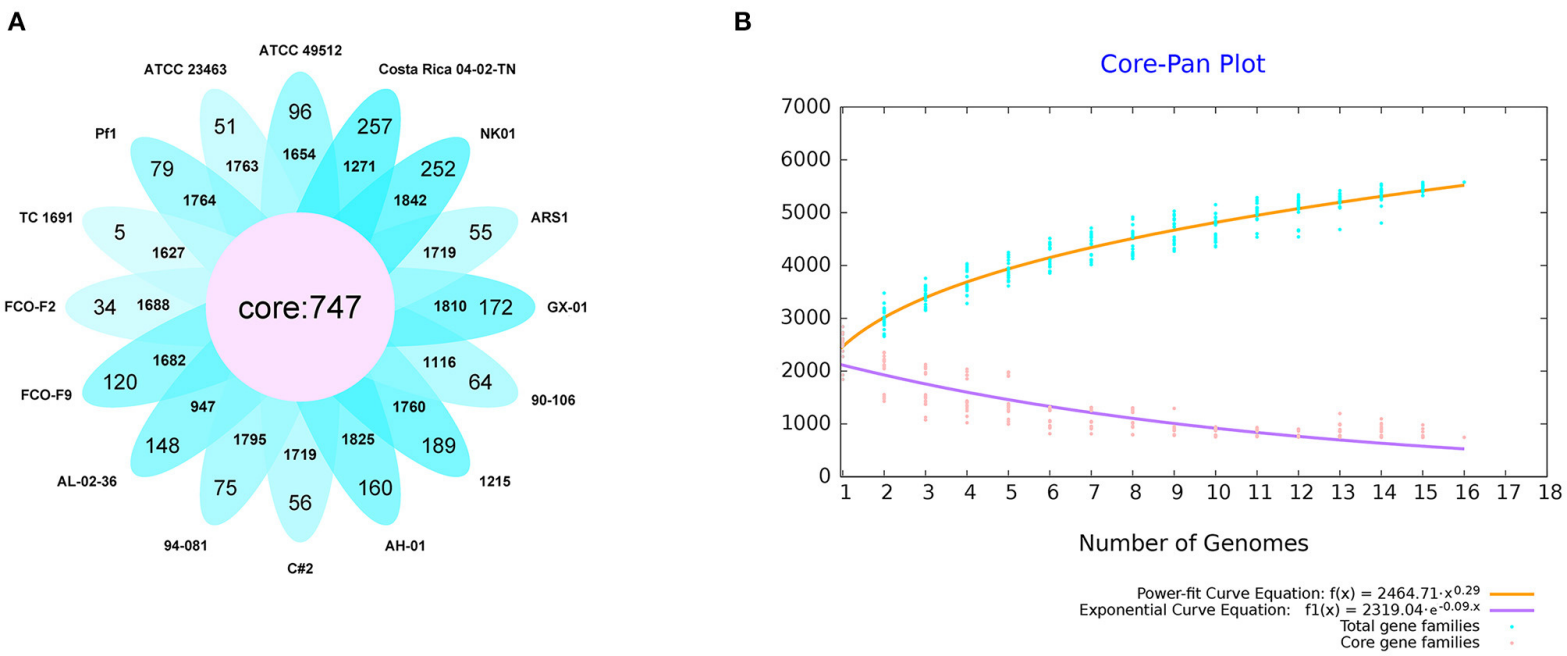
Results of the core pan-genome from 16 isolates of *F. columnare*. **(A)** Diagram of predicted core genes, accessory genes (the number close to the middle), and unique genes (the number close to the outside of the petals). **(B)** Core pan-genome curves of exponential and power-fit are demonstrated by yellow and purple lines, respectively. The blue dots represent total gene families, and the pink ones are core gene families. The equation of core and pan-genome is shown at the bottom of the figure.

We first performed core genome predictions of the 16 *F. columnare* strains. All core genes (core genome) and accessory genes in a species comprise the pan-genome. Core genes are homologous genes that exist within a species, while unique genes only exist in a certain bacterial strain. Core and unique genes are generally used as the research basis for identifying functional differences between strains. We identified 747 core genes, some accessory genes, and unique genes in the gene repertoire of 16 *F. columnare* strains; these are displayed in a petal diagram in Figure 6A. The numbers close to the middle of the petals represented accessory genes, and the numbers near the outside represented unique gene numbers. The number of core genes was significantly lower than that of other bacteria. The number of accessory genes in these strains was approximately the same, ranging between 947 and 1,842. Interestingly, when we only analyzed the genomes of genetic groups 1, 2, and 3 (not including genetic group 4), the number of core genes was 1,837. Exponential and power-fit equations of the core genome and pan-genome were used to infer changes after adding a new genome. With each addition of a new genome, the pan-genome gene repertoire increased gradually, but the core genome size reduced progressively (Figure 6B). The expected size of the pan-genome was 5,578, with an expansion rate of *b* = 0.290709, which meant that the pan-genome was still open but may be closed soon (Costa et al., 2020). For the core genome, the estimated size was 528.01, and the exponential decay model was f1(x) = 2,319.04^*^e∧(−0.09.x).

#### 3.3.2. cgMLST

**Figure 7:**
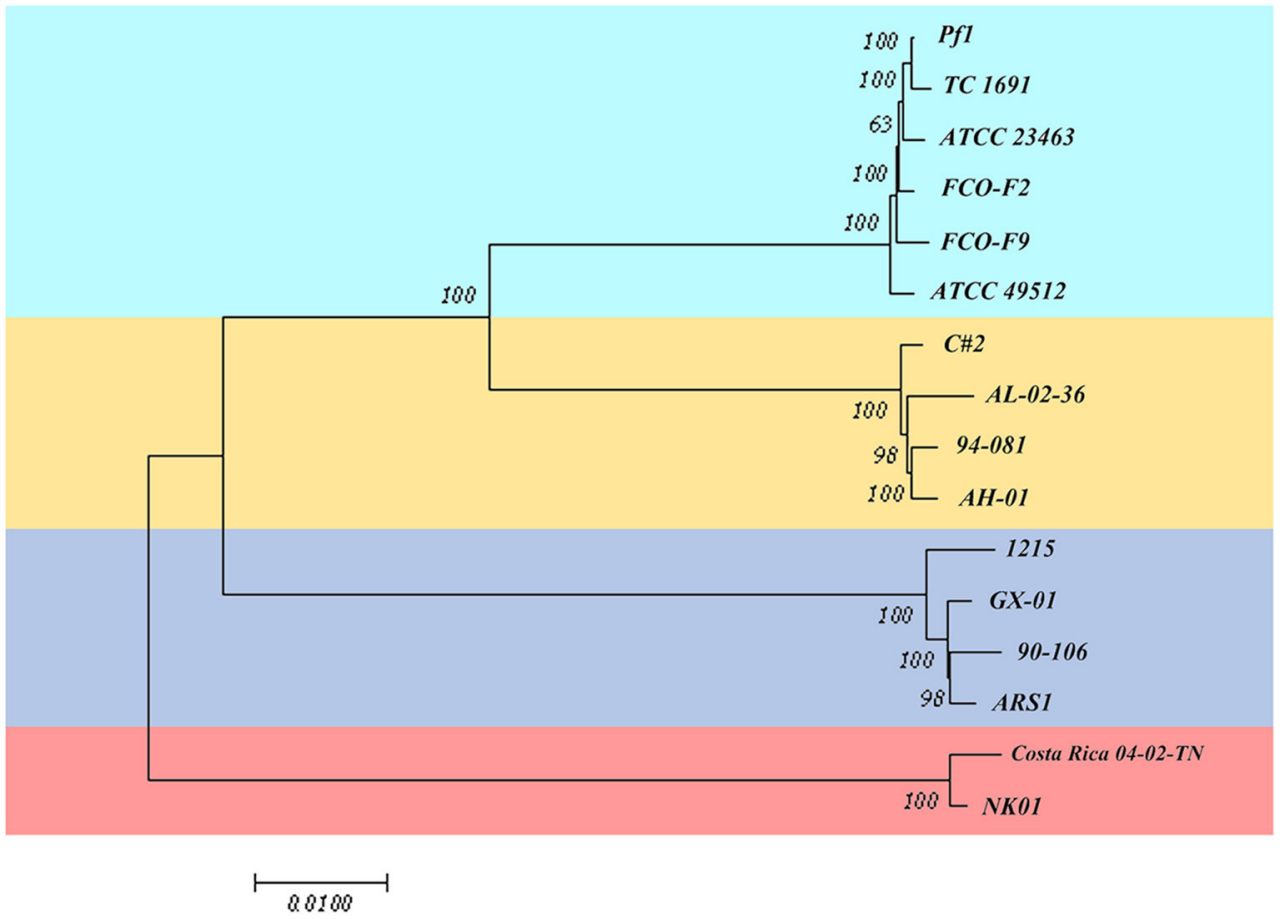
Multilocus sequence typing scheme based on the core gene sequence of 16 isolates of *F. columnare*.

Based on the core gene sequence retrieved above, we designed an MLST scheme. As shown in Figure 7, the strains were classified into four sequence types, which corresponded to the four genetic groups. However, the classification was different from the results in Figure 1. Strains of genetic groups 1, 2, 3, and 4 were divided into two large clades. In the first clade, genetic groups 1, 2, and 3 were further divided into two clusters. Finally, genetic group 1 and 2 strains were separated into two groups. Genetic groups 1 and 2 had the closest relationship, followed by genetic group 3, while the strains of genetic group 4 had the most distant relationship with them.

#### 3.3.3. Genome alignment

**Figure 8:**
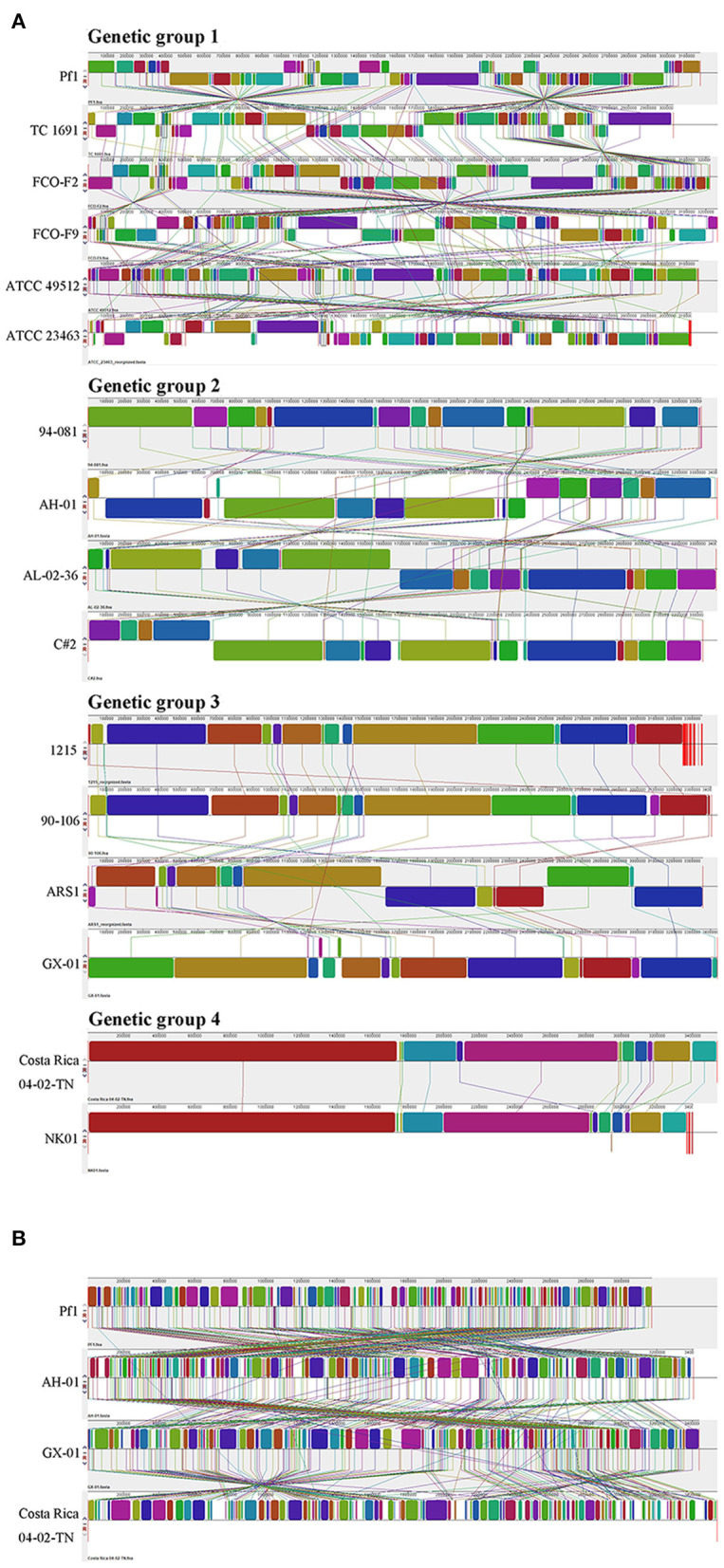
Genome alignment of *F. columnare* conducted by MAUVE. **(A)** A separate comparison of 16 genomes from three genomovars (four genetic groups). **(B)** Strains Pf1, AH-01, and GX-01, which were isolated from China, and strain Costa Rica 04-02-TN are compared as representatives for combined analysis.

To further explore the differences among strains, we performed genome alignment using MAUVE software, which can identify potential horizontal transfer loci and genomic rearrangements. ATCC 23463, NK01, 1215, and ARS1 were rearranged with Medusa (Bosi et al., 2015); information concerning the reorganized genomes is provided in Supplementary Table 6. When strains belonging to the same genetic group were compared together, some local gene clusters synchronized across the genome, but some gene inversions (denoted by the regions below the horizontal line) and rearrangements (denoted by the colored lines) still existed. Figure 8A depicts four pictures; each shows the genome syntenic relationship of strains in the different genetic groups. Genome alignment revealed a large number of transpositions and rearrangements in the genomes of strains in genetic group 1. Among them, the TC 1691 and FCO-F2 pair of genomes were the most syntenic, with only a small range of inversions between them. Different degrees of rearrangement also occurred in the strains of genetic groups 2, 3, and 4; generally, there were more syntenic regions in those groups than in genetic group 1. Genetic group 4 strains seemed to have the fewest local collinear blocks with minimal rearrangement among these four groups, which may be related to the lower number of samples. When we compared strains Pf1, AH-01, and GX-01 with Costa Rica 04-02-TN, which represented the four genetic groups (and also three genomovars), we observed extensive translocations, inversions, and rearrangements to a greater extent than those of intragroup comparisons (Figure 8B).

#### 3.3.4. Genome-wide orthology and phylogenetic analysis

**Figure 9:**
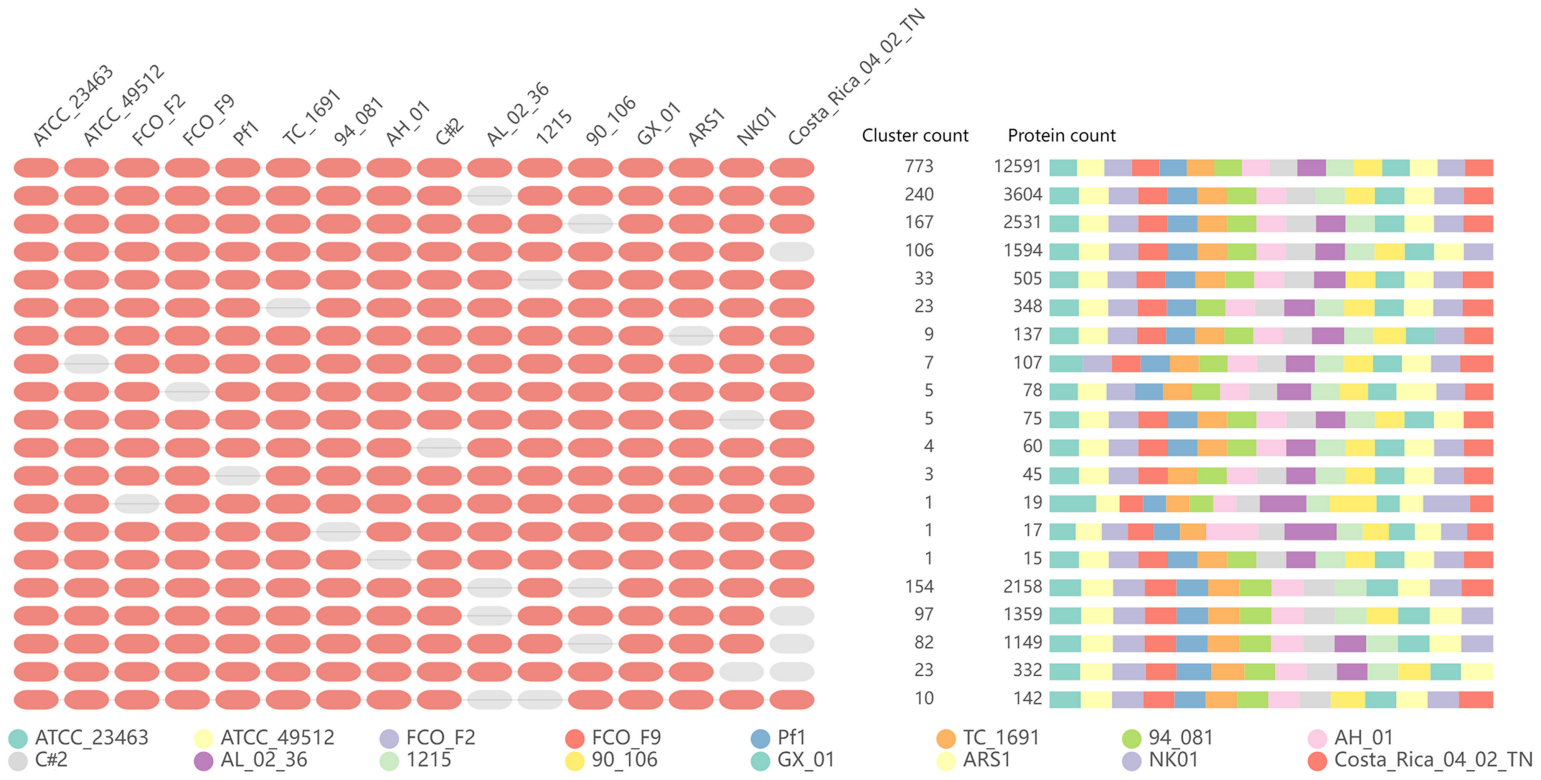
Summary graph of the overlapping orthologous gene clusters (≥14) across multiple strains.

In addition, we performed orthology analysis using the default parameters of OrthoVenn2, which generated clusters of proteins, each consisting of orthologs or paralogs among species. Figure 9 shows the number of overlapping clusters (≥14) shared between the 16 *F. columnare* isolates. All strains formed a total of 4,039 clusters, 3,333 orthologous clusters (at least two strains), 773 orthologous clusters containing all isolates tested, and 706 single-copy gene clusters. The details of single-copy clusters are provided in Supplementary Table 7.

**Figure 10:**
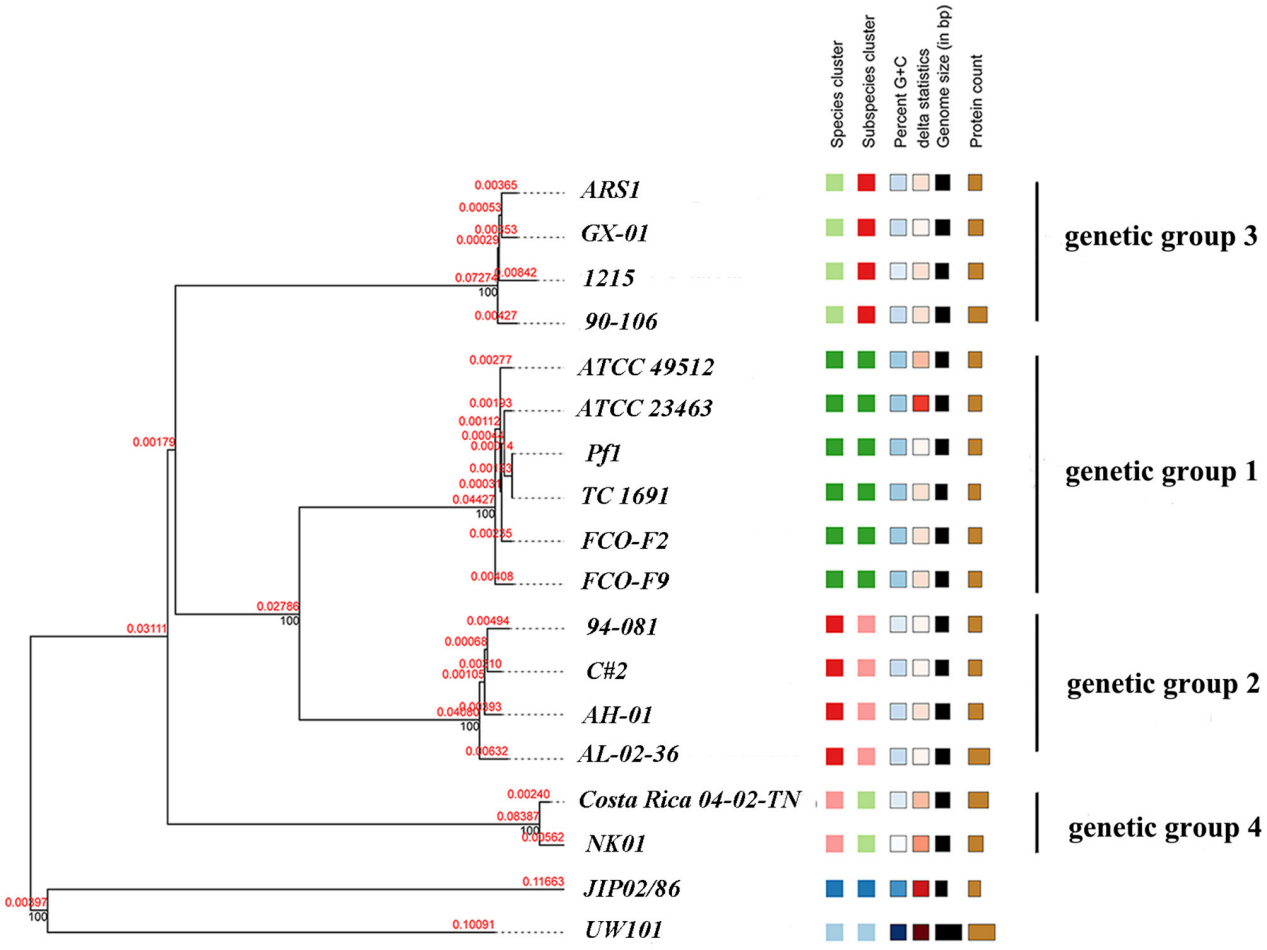
Phylogenetic tree of 16 *F. columnare* isolates with *F. johnsoniae* UW101 and *F. psychrophilum* JIP02/86 as outgroup clades. The phylogenic tree was constructed using TYGS with the modified neighbor-joining method (FastME) based on the alignment of whole-genome sequences. Red numbers represent branch length values, which are scaled in terms of the GBDP distance formula d5. Black numbers are GBDP pseudo-bootstrap support values from 100 replications.

A whole-genome phylogenetic tree of 18 isolates was reconstructed using the TYGS pipeline. The phylogenetic tree consisted of two primary branches composed of *F. columnare* and the other *Flavobacterium* outgroup clades (Figure 10). Among the *F. columnare* strains, two main branches included isolates of genetic group 4 and a large branch consisting of three other clades. The findings implied that genetic group 4 was very distant from the others. Genetic group 1 and 2 strains clustered in a subclade, while genetic group 3 strains clustered in another subclade. Genetic groups 1 and 2 were also divided into two major groups. The clustering of strains from the four genetic groups into four subgroups supported the classification by the cgMLST method above. Notably, genetic groups 1 and 2 had a close relationship, showing a less close relationship with group 3 and a more distant relationship with group 4.

#### 3.3.5. ANI and dDDH analyses

**Figure 11:**
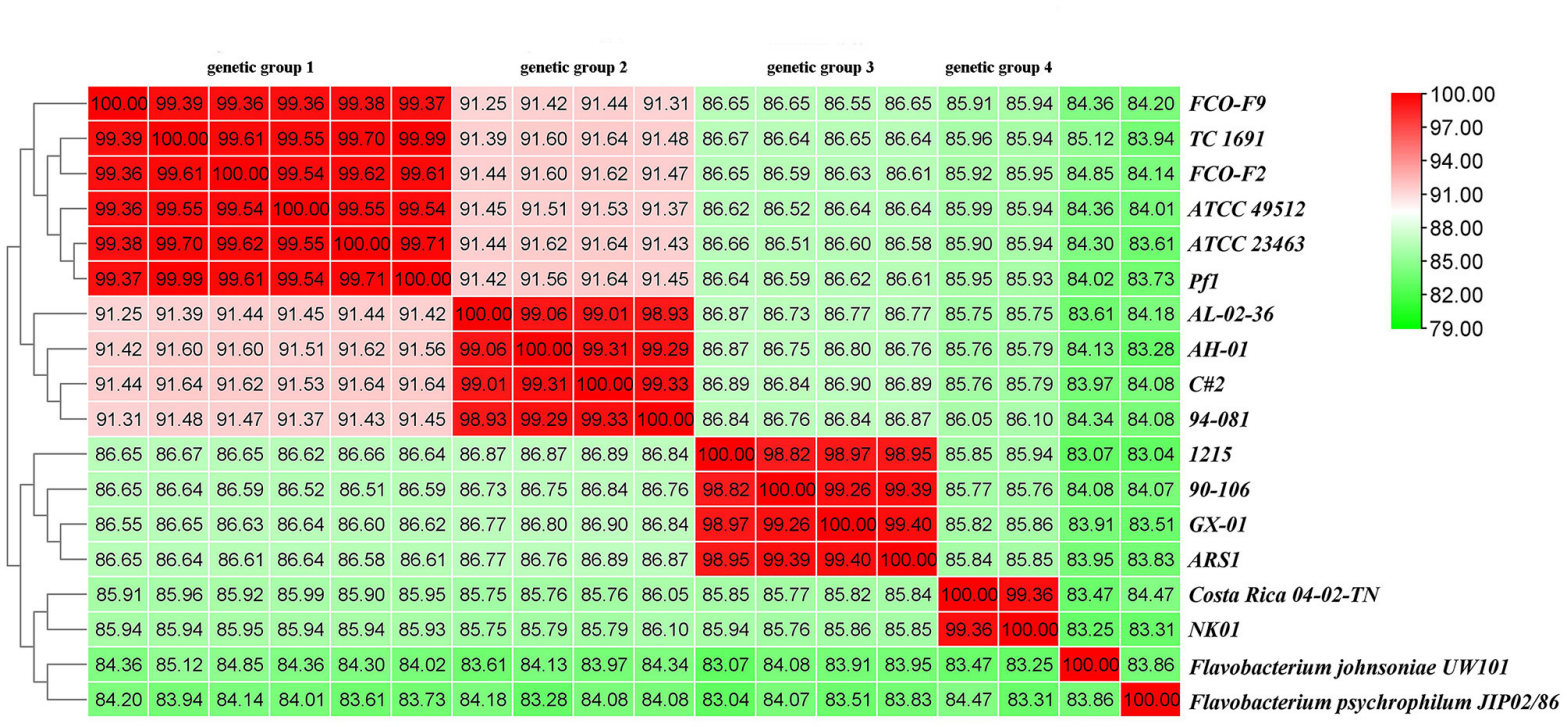
Average nucleotide identity (ANI) similarities among 16 *F. columnare* isolates, *F. johnsoniae* UW101, and *F. psychrophilum* JIP02/86.

Finally, the ANI was determined. ANI is an index comparing genetic relationships between genomes at the nucleotide level. ANI values can support the results of the phylogenetic tree. The ANI values of *F. columnare* and *F. johnsoniae* UW101 were between 83.07 and 85.12%, while those between *F. columnare* and *F. psychrophilum* JIP02/86 were 83.04% to 84.47% (Figure 11). Cluster analysis revealed that strains of genetic groups 3 and 4 were more closely clustered with strains of *F. johnsoniae* and *F. psychrophilum*. There were four levels of ANI values for *F. columnare* strains, classifying these strains into four groups, consistent with the results of the phylogenetic tree. Intraspecific ANI values for *F. columnare* genetic group 1 (genomovar I) ranged between 99.36% and 100%, those for genetic group 2 (genomovar II) ranged from 98.93% to 100%, those for genetic group 4 (genomovar II) were 99.36% to 100%, and those for genetic group 3 (genomovar III) were between 98.82% and 100%. In contrast, values between each pair of groups were as follows: 91.25%−91.64% (genetic group 1 vs. genetic group 2), 86.61%−86.67% (1 vs. 3), 85.90%−85.99% (1 vs. 4), 86.73%−86.90% (2 vs. 3), 85.75%−86.10% (2 vs. 4), and 85.76%−85.94% (3 vs. 4).

In addition, the dDDH value was also determined using GGDC. Consistent with the ANI results, these strains were divided into four groups. The intragroup dDDHs of genetic groups 1 and 4 exceeded 93%, and those of genetic groups 2 and 3 were >88%. The calculated dDDH value between genetic groups 1 and 2 was approximately 43%, between groups 1 and 3 was approximately 30%, and between groups 1 and group 4 was ~28%. The numerical boundaries between genetic groups were obvious and lower than the most promising DDH threshold to delineate species (Supplementary Table 8).

## 4. Discussion

*F. columnare* is an important fish pathogen that poses substantial risks to production. In recent years, many reports have chronicled large-scale deaths of farmed fish caused by *F. columnare*. This bacterium has a wide host range and a large geographical distribution, which may result in relatively high genetic diversity in *F. columnare* populations. In this study, we performed a genetic analysis of strains isolated by our laboratory, characterized the genomes of two Chinese isolates, and performed a comparative genomic analysis of available genomes from different regions globally. Our goal was to deepen our understanding of this pathogen and reveal the relationship between its genetic heterogeneity and molecular phylogenetics.

For years, many studies have addressed the genetic diversity and genotyping methods of *F. columnare*. To date, there are two widely accepted genotyping methods. One divides *F. columnare* into three main genomovars based on 16S-RFLP. The other divides *F. columnare* into four genetic groups based on MLPA analysis. The correspondence between the two approaches is as follows: genomovar I corresponds to genetic group 1, genomovar III corresponds to genetic group 3, and genomovar II is composed of genetic groups 2 and 4. Therefore, we first performed a genetic analysis on 55 *F. columnare* strains that were previously isolated. The results revealed that these strains could be classified into four groups, which corresponded to the four genetic groups identified by the previous method. The similarity of 16S rRNA gene sequences served as a common criterion for bacterial classification. The well-accepted criterion of 16S rRNA gene sequence identity for the rank of species was a threshold value of approximately 97%−99% (Stackebrandt, 2006; Tindall et al., 2010; Meier-Kolthoff et al., 2013). Based on this criterion, these 55 strains seemed to belong to four species. However, in many cases, it was hard to differentiate two species using 16S rRNA gene sequences alone, as some species shared a high level of 16S rRNA gene sequence similarity (>99 %), even though they were separated by DDH (Rosselló-Mora and Amann, 2001). Like the 16S rRNA gene, housekeeping genes are important and ubiquitous in bacteria. They have evolved more rapidly than 16S rRNA genes and can therefore be used to distinguish recently diverged lineages. Several genes, including *gyrA, gyrB, rpoB*, and *tuf*, can serve as markers for microbial diversity (Case et al., 2007; Ghebremedhin et al., 2008; Poirier et al., 2018; Liu et al., 2022). Accordingly, we also constructed the phylogenetic tree using *gyrB, tuf*, and *dnaK* gene sequences that were consistent with previously published results, while the multilocus tree and 16S rRNA gene tree are not completely the same (LaFrentz et al., 2018). The 16S rRNA gene tree was consistent with the multilocus tree, which also proved that the 55 strains isolated from China covered all known types. Combined with the results of the phylogenetic analysis, the four genetic groups (three genomovars) were displayed more clearly. Although we reconstructed the phylogenetic relationships of *F. columnare* strains based on 16S rRNA single and housekeeping gene sequences, they were hardly considered true genome-scale phylogenetic methods and may not definitively resolve evolutionary relationships within many groups (Castillo et al., 2016; Kayansamruaj et al., 2017; LaFrentz et al., 2018).

To further investigate the genomic diversity and evolution of *F. columnare*, we decided to perform whole-genome sequencing and comparative genome analysis. This allows us to examine the genetic variations and phylogenetic relationships among the different strains at a higher resolution than with single-gene or multilocus methods. The difference in genome size and composition between AH-01 and GX-01 may simply be inferred as a variation. Genome size, coding sequence number, RNA number, and G+C content are very similar to those of other published *F. columnare* genomes (Kayansamruaj et al., 2017). In prokaryotic genomes, plasmids are genetic elements for colonization and replication; they are believed to be a major driving force of prokaryote evolution as they can migrate between populations to induce lateral DNA transfer (Wein and Dagan, 2020). To date, no plasmids have been reported in *F. columnare*, except for the circular plasmid identified here in AH-01, highlighting the importance of this study and whole-genome sequencing for bacterial pathogens. In the plasmid of AH-01, we found seven GIs, which contained some transcriptional regulators. Additionally, protease, penicillin-binding protein, and drug efflux transporter were also found, which may also support the degree of resistance of AH-01 to antibiotics (data not shown).

Bacteriophages (phages) are the most abundant biological entities on earth and are recognized as a major contributor to microbial genetic variation and diversity (Fortier and Sekulovic, 2013). AH-01 and GX-01 possess incomplete phage elements such as transposases and many phage-like proteins. GIs are part of the flexible bacterial gene pool, and a wide variety of GIs are intimately related to phage- or plasmid-derived sequences, including transfer genes or integrases, through their evolutionary origins (Hacker and Carniel, 2001; Juhas et al., 2009). Transmissible genomic elements, such as transposase and integrase, were found in AH-01 and GX-01. Moreover, the vast majority of these regions contained hypothetical genes, suggesting that these possible GIs may have been acquired horizontally. The GI numbers of AH-01 and GX-01 were in the range of those identified in previous studies (5–29 GIs per genome), while a 43-kb GI shared among other *F. columnare* strains was not present in these two strains (Kayansamruaj et al., 2017; Tekedar et al., 2017), which may imply a high level of gene exchange and genome plasticity in *F. columnare* strains, as well as the influence of geographic location on genome evolution.

In addition to prophages and GIs, prokaryotic clustered regularly interspaced short palindromic repeats (CRISPRs) and CRISPR-associated (Cas) proteins in prokaryotic genomes constitute a bacterial adaptive immune system against foreign nucleic acids such as those of bacteriophages or plasmids. CRISPR arrays can store foreign DNA using short DNA spacers (Amitai and Sorek, 2016). In this study, three distinct CRISPR systems were identified in *F. columnare* isolates AH-01 and GX-01, which also carried cas enzyme genes (4 and 1, respectively) that catalyzed the production of spacer sequences. The CRISPR systems of *F. psychrophilum* contain spacers that match bacteriophage 6H (Castillo et al., 2016). Conversely, the *F. columnare* AH-01 and GX-01 CRISPR systems did not match any known sequences. However, many functional proteins were identified, including transposase, thiamine phosphate synthase, CDI toxins, and others. This might be because of their unique ecological environments and greater pressure from other foreign DNA.

To understand the evolutionary history and diversity of *F. columnare*, we analyzed the core and accessory genomes of these strains. The genetic material of prokaryotes is inherited asexually from ancestral cells. The accumulation of mutations during this cloning process leads to the generation of subpopulations with selective advantages. Bacterial species maintain a “genetic pool” much larger than that in each strain. Each pool has a conserved set of core genes and some accessory genes (Mathee et al., 2008). Core genomes are believed to represent bacterial taxa at different taxonomic levels. Their components can be used to trace the evolutionary history of clonal lineages (Hacker and Carniel, 2001; Lefebure and Stanhope, 2007). The core genome of all *F. columnare* strains tested contained 747 genes, corresponding to approximately 25% of the genome, which represents a very small proportion of this core genome. For comparison, the proportion of *Escherichia coli* and *Flavobacterium psychrophilum* is approximately 60% and 73%, respectively (Vieira et al., 2011; Castillo et al., 2016).

In addition to core genes encoding basic metabolic functions, bacterial genomes also contain a variable number of accessory genes that may have been acquired by horizontal gene transfer (Schmidt and Hensel, 2004). The accessory genes often appear to move laterally between strains, forming new trait combinations, and may encode adaptive traits beneficial to bacteria for adapting to certain environmental growth conditions (Segerman, 2012). However, the number of samples analyzed currently is still relatively small. Studies involving larger sample sizes are needed in future to delineate the characteristics of the pan-genome and the evolutionary state of the *F. columnare* population.

Sequence similarity within core genomes is considered one of the best phylogenetic metrics for comparing microbial genomes (Rokas et al., 2003). Recently, an efficient, accurate, and reproducible cgMLST method was proposed for whole-genome sequencing-based strain differentiation and epidemiological investigation and has been applied to *E. coli* (Mellmann et al., 2011), *Listeria monocytogenes* (Schmid et al., 2014), *Mycobacterium tuberculosis* (Kohl et al., 2014), *Enterococcus faecium* (De Been et al., 2015), and other bacteria. To compare the phylogenetic relationships among the strains based on their core genomes, we used this method. Undoubtedly, the evolutionary relationship between genetic groups 1 and 2 was closer than that between the other two genetic groups. However, concerning the genetic relationship with genetic groups 3 and 4 differed according to cgMLST and single-gene phylogenetic tree analyses. These findings indicated that the existing *F. columnare* population may need to be further classified.

In addition to horizontal gene transfer, the genomes of bacterial species can evolve through a variety of processes, including mutations or rearrangements (Schmidt and Hensel, 2004). To examine the genomic structure and rearrangements among strains, we performed genome alignment using MAUVE. Genetic group 1 featured more data and diversity than the other groups. Large genomic differences were evident among the different genetic groups, including inversions, translocations, and rearrangements, with few regions of collinearity. These differences might be a factor in adaptation to new hosts, antibiotics, or living environments and may imply that the present *F. columnare* is not a single species but rather a collection of multiple species.

Another method we used to compare the genomes was orthology analysis. Orthologs are genes diverged by a speciation event. The concept of orthology between genes is the core of any comparative genomic analysis for estimating species phylogeny (Koonin et al., 2000). Orthology analysis determines whether a pair of homologous genes are orthologs (derived from speciation) or paralogs (derived from gene duplication) and can infer the function of other genes that are orthologously related to the gene that is already known (Gogarten and Townsend, 2005). We used GBDP for phylogenetic analyses. Our results revealed two main branches: a large branch consisting of three other clades and genetic group 4 as a single clade, similar to the cgMLST result and previous research (LaFrentz et al., 2018). Strains from genetic groups 1, 2, and 3 formed three distinct subgroups, but the strains from genetic groups 1 and 2 also grouped together in a larger clade, separate from group 3. Based on the results of phylogenetic analysis and genome alignments, we suggested that genetic groups 1 and 2 were closer to each other and further from the other two groups in evolutionary distance.

The results of phylogenetic analysis and the genome alignments supported the view that the four groups were distant from each other in their evolutionary distance. ANI and dDDH values were determined to explore this idea. The rank of species was restricted by a combination of well-accepted criteria, which include 16S rRNA gene sequence identities, with a threshold of approximately 98.7%, ANI with thresholds of approximately 94–96%, and DDH with a threshold of approximately 70% (Yarza et al., 2014). DDH has long been considered the gold standard for bacterial species circumscriptions. Currently, ANI is the most robust measurement for species demarcation in prokaryotes. The ANI value of approximately 94% corresponds to the traditional 70% DNA–DNA standard (Richter and Rossello-Mora, 2009; Kim et al., 2014). However, we observed that the ANI values between different genetic groups were below the standard threshold (similarly reflected in dDDH results), which further supports the results of the phylogenetic analysis mentioned earlier.

Based on the findings presented, we propose that *F. columnare*, currently considered a single species, should be considered a collection of multiple species and needs to be reclassified. A revision of the species designation of *F. columnare* has been previously proposed, based on the detection of quantitative differences in fatty acid profiles and matrix-assisted laser desorption/ionization time of flight mass spectrometry analysis (Cai, 2018; LaFrentz et al., 2022). Our results were generally consistent with the previous findings. Although the genetic relationship between genetic groups 1 and 2 was closer than that between the other two genetic groups, the ANI value between them did not reach the 98% standard for distinguishing subspecies. This suggested high genetic diversity within the species, with time and geographical evolution, as well as lateral gene transfer and gene loss with the division into new species that may become increasingly different over time. However, the ANI values within different groups were also at the boundary threshold, which may suggest that the geographical distribution of different isolates influences their genetic differentiation. Therefore, more new species may be discovered as more genomes become available for scrutiny.

In conclusion, this study provided the complete genomes of AH-01 and GX-01 and the genetic analysis of *F. columnare*, which provided valuable data for subsequent analyses. We demonstrated a high level of gene variation and horizontal transfer, which were the basis of genetic diversity, genome plasticity, and functional evolution in different environments. Furthermore, our analyses of core pan-genomes, cgMLST, MAUVE alignment, ANI, and dDDH supported the notion that the currently existing *F. columnare* was an assemblage of several distinct species. The levels of divergence between these species were at least equivalent to those between recognized bacterial species. Overall, our results provided valuable data for subsequent analysis greatly expanded our knowledge of evolutionary events in *F. columnare* and suggested a new taxonomic perspective.

## Data availability statement

The datasets presented in this study can be found in online repositories. The names of the repository/repositories and accession number(s) can be found in the article/Supplementary material.

## Author contributions

RH: methodology, validation, data curation, and writing—original draft. YH, RX, WG, and ZL: validation and resources. MZ: data curation. YL and XD: conceptualization, project administration, and writing-review and editing. All authors contributed to the article and approved the submitted version.
