# Supplementary material for: Genomic evidence of genetic diversity and functional evolution in Flavobacterium columnare
Source: Front Microbiol. 2023 Sep 28;14:1240471. doi: 10.3389/fmicb.2023.1240471 (PMC10568018; doi:10.3389/fmicb.2023.1240471)
Supplement: Supplementary file 9 [file Image_1.PDF]

A

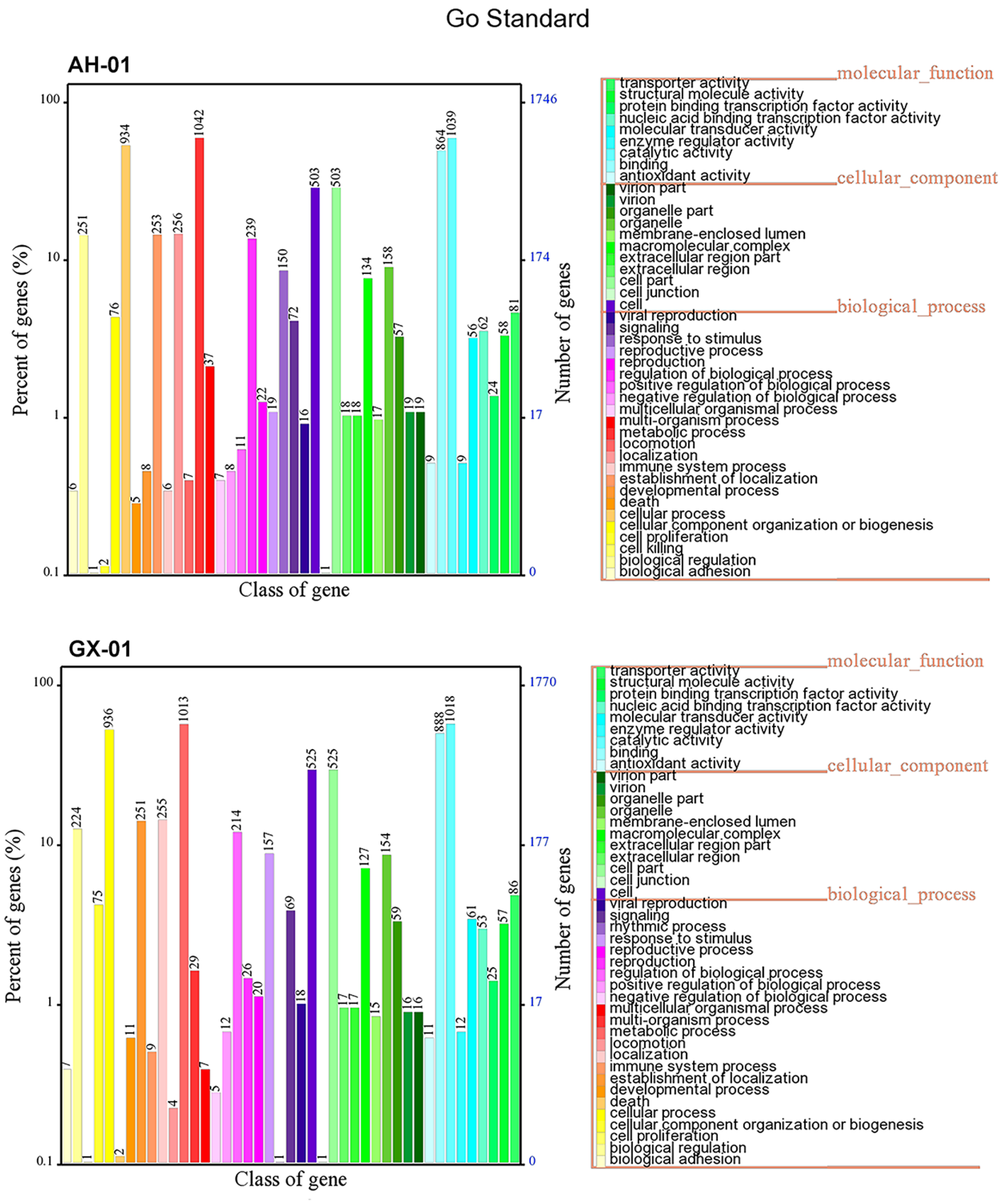
Go Standard
AH-01
Percent of genes (%)
Number of genes
Class of gene
molecular_function
transporter activity
structural molecule activity
protein binding transcription factor activity
nucleic acid binding transcription factor activity
molecular transducer activity
enzyme regulator activity
catalytic activity
binding
antioxidant activity
cellular_component
virion part
virion
organelle part
organelle
membrane-enclosed lumen
macromolecular complex
extracellular region part
extracellular region
cell part
cell junction
cell
biological_process
viral reproduction
signaling
response to stimulus
reproductive process
reproduction
regulation of biological process
positive regulation of biological process
negative regulation of biological process
multicellular organismal process
multi-organism process
metabolic process
locomotion
localization
immune system process
establishment of localization
developmental process
death
cellular process
cellular component organization or biogenesis
cell proliferation
cell killing
biological regulation
biological adhesion
GX-01
Percent of genes (%)
Number of genes
Class of gene
molecular_function
transporter activity
structural molecule activity
protein binding transcription factor activity
nucleic acid binding transcription factor activity
molecular transducer activity
enzyme regulator activity
catalytic activity
binding
antioxidant activity
cellular_component
virion part
virion
organelle part
organelle
membrane-enclosed lumen
macromolecular complex
extracellular region part
extracellular region
cell part
cell junction
cell
biological_process
viral reproduction
signaling
rhythmic process
response to stimulus
reproductive process
reproduction
regulation of biological process
positive regulation of biological process
negative regulation of biological process
multicellular organismal process
multi-organism process
metabolic process
locomotion
localization
immune system process
establishment of localization
developmental process
death
cellular process
cellular component organization or biogenesis
cell proliferation
biological regulation
biological adhesion

B

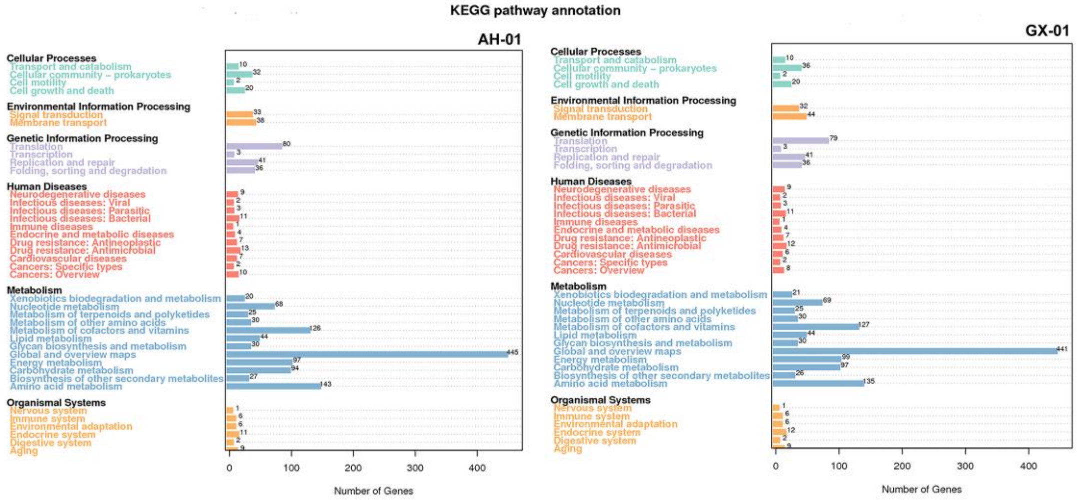
KEGG pathway annotation
AH-01
GX-01
Cellular Processes
Transport and catabolism
Cellular community – prokaryotes
Cell motility
Cell growth and death
Environmental Information Processing
Signal transduction
Membrane transport
Genetic Information Processing
Translation
Transcription
Replication and repair
Folding, sorting and degradation
Human Diseases
Neurodegenerative diseases
Infectious diseases: Viral
Infectious diseases: Parasitic
Infectious diseases: Bacterial
Immune diseases
Endocrine and metabolic diseases
Drug resistance: Antineoplastic
Drug resistance: Antimicrobial
Cardiovascular diseases
Cancers: Specific types
Cancers: Overview
Metabolism
Xenobiotics biodegradation and metabolism
Nucleotide metabolism
Metabolism of terpenoids and polyketides
Metabolism of other amino acids
Metabolism of cofactors and vitamins
Lipid metabolism
Glycan biosynthesis and metabolism
Global and overview maps
Energy metabolism
Carbohydrate metabolism
Biosynthesis of other secondary metabolites
Amino acid metabolism
Organismal Systems
Nervous system
Immune system
Environmental adaptation
Endocrine system
Digestive system
Aging
Number of Genes

C

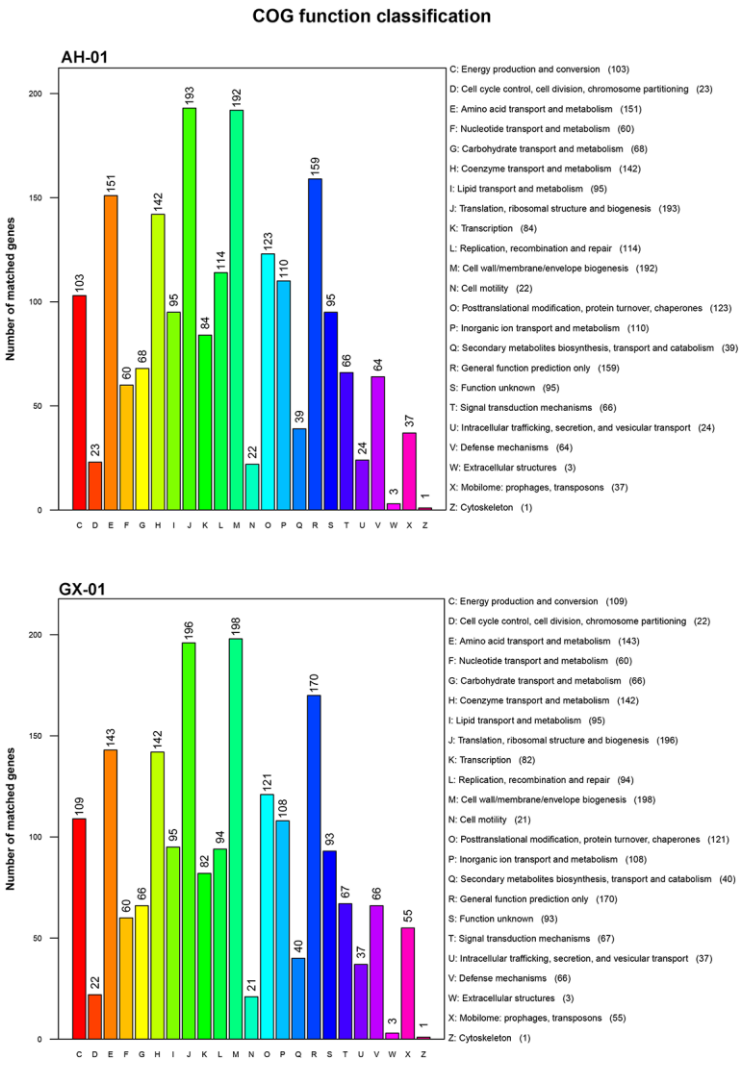
COG function classification
AH-01
GX-01
Number of matched genes
C: Energy production and conversion (103)
D: Cell cycle control, cell division, chromosome partitioning (23)
E: Amino acid transport and metabolism (151)
F: Nucleotide transport and metabolism (60)
G: Carbohydrate transport and metabolism (68)
H: Coenzyme transport and metabolism (142)
I: Lipid transport and metabolism (95)
J: Translation, ribosomal structure and biogenesis (193)
K: Transcription (84)
L: Replication, recombination and repair (114)
M: Cell wall/membrane/envelope biogenesis (192)
N: Cell motility (22)
O: Posttranslational modification, protein turnover, chaperones (123)
P: Inorganic ion transport and metabolism (110)
Q: Secondary metabolites biosynthesis, transport and catabolism (39)
R: General function prediction only (159)
S: Function unknown (95)
T: Signal transduction mechanisms (66)
U: Intracellular trafficking, secretion, and vesicular transport (24)
V: Defense mechanisms (64)
W: Extracellular structures (3)
X: Mobilome: prophages, transposons (37)
Z: Cytoskeleton (1)
C: Energy production and conversion (109)
D: Cell cycle control, cell division, chromosome partitioning (22)
E: Amino acid transport and metabolism (143)
F: Nucleotide transport and metabolism (60)
G: Carbohydrate transport and metabolism (66)
H: Coenzyme transport and metabolism (142)
I: Lipid transport and metabolism (95)
J: Translation, ribosomal structure and biogenesis (196)
K: Transcription (82)
L: Replication, recombination and repair (94)
M: Cell wall/membrane/envelope biogenesis (198)
N: Cell motility (21)
O: Posttranslational modification, protein turnover, chaperones (121)
P: Inorganic ion transport and metabolism (108)
Q: Secondary metabolites biosynthesis, transport and catabolism (40)
R: General function prediction only (170)
S: Function unknown (93)
T: Signal transduction mechanisms (67)
U: Intracellular trafficking, secretion, and vesicular transport (37)
V: Defense mechanisms (66)
W: Extracellular structures (3)
X: Mobilome: prophages, transposons (55)
Z: Cytoskeleton (1)

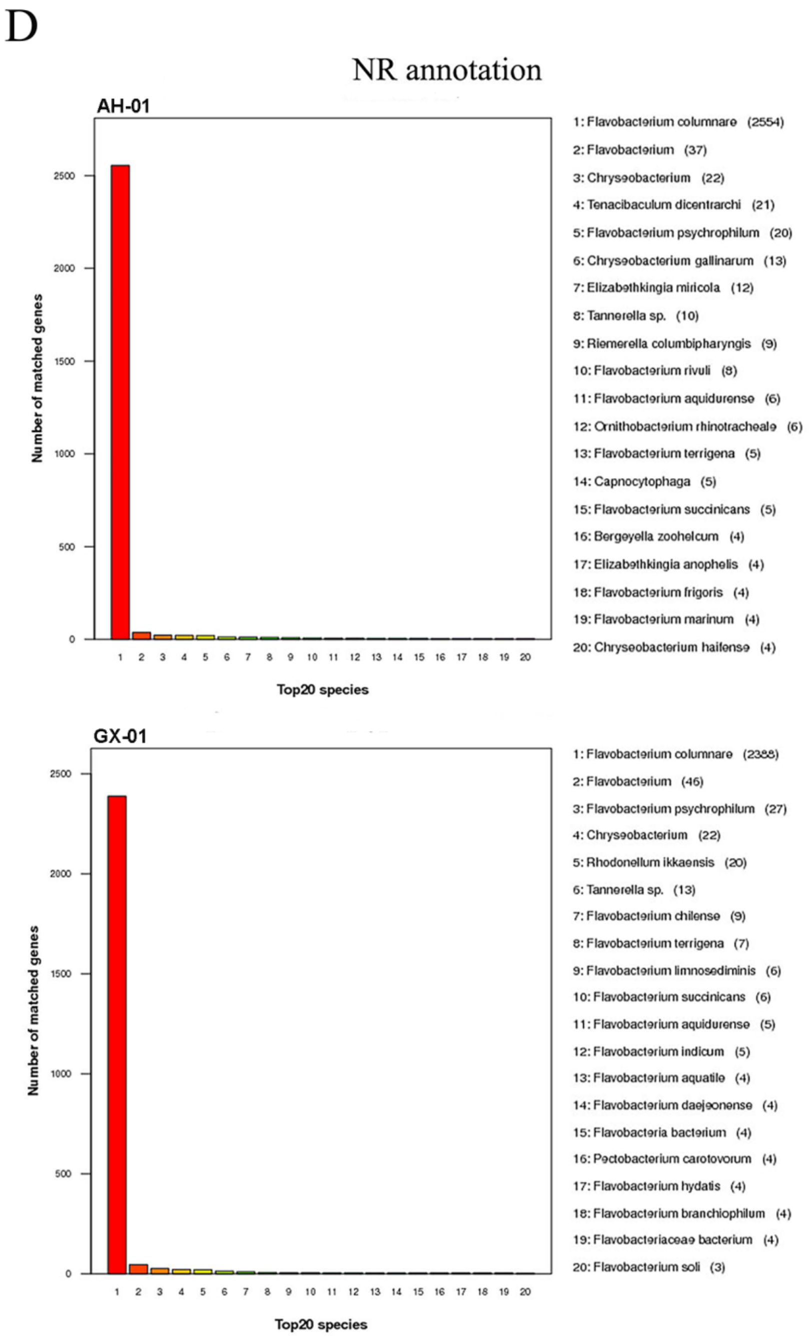

SFig. 1. *F. columnare* AH-01 and GX-01 genome annotated by GO (A), KEGG (B), COG (C) and NR (D) database.
